# Multifunctional Nanoparticles as Radiosensitizers to Overcome Hypoxia-Associated Resistance in Cancer Radiotherapy

**DOI:** 10.3390/nano15010037

**Published:** 2024-12-29

**Authors:** Ming-Hong Chen, Hon-Pan Yiu, Yu-Chi Wang, Tse-Ying Liu, Chuan Li

**Affiliations:** 1 Division of Neurosurgery, Department of Surgery, Far Eastern Memorial Hospital, New Taipei City 220216, Taiwan; chen.minghong@gmail.com; 2 Department of Electrical Engineering, Yuan Ze University, Taoyuan City 320315, Taiwan; 3 Department of Biomedical Engineering, National Yang Ming Chiao Tung University, Taipei 112304, Taiwan; yiuhonpan@gmail.com (H.-P.Y.); tomato13590@gmail.com (Y.-C.W.)

**Keywords:** hypoxia, oxygen fixation, radiotherapy, intracellular Fenton reaction, chemodynamic therapy

## Abstract

Hypoxia, a phenomenon that occurs when the oxygen level in tissues is lower than average, is commonly observed in human solid tumors. For oncological treatment, the hypoxic environment often results in radioresistance and chemoresistance. In this study, a new multifunctional oxygen carrier, carboxymethyl hexanoyl chitosan (CHC) nanodroplets decorated with perfluorohexane (PFH) and superparamagnetic iron oxide (SPIO) nanodroplets (SPIO@PFH-CHC), was developed and investigated. PFH-based oxygen carriers can augment oxygenation within tumor tissues, thereby mitigating radioresistance. Concurrently, oxygenation can cause deoxyribonucleic acid (DNA) damage via oxygen fixation and consequently suppress cancer cell proliferation. Moreover, these pH-sensitive nanodroplets allow higher cellular uptake with minimal cytotoxicity. Two distinctive mechanisms of SPIO@PFH-CHC nanodroplets were found in this study. The SPIO nanoparticles of the SPIO@PFH-CHC nanodroplets can generate hydroxyl radicals (HO^•^) and other reactive oxygen species (ROS), which is vital to chemodynamic therapy (CDT) via the Fenton reaction. Meanwhile, the higher X-ray absorption among these nanodroplets leads to a local energy surge and causes more extensive deoxyribonucleic acid (DNA) damage via oxygen fixation. This study demonstrates that low cytotoxic SPIO@PFH-CHC nanodroplets can be an efficient radiosensitizer for radiation therapy.

## 1. Introduction

Radiation therapy (RT), one of the common treatments for cancer, utilizes high-energy radiation (X-rays or γ-rays) to ionize molecules and cause direct or indirect DNA damage [1]. Such damages are more fatal for cancer cells than normal cells because cancer cells usually bank on their high proliferation rate. However, one disadvantage of radiation therapy is that its modality is not highly specific. Exposure of normal tissues along the pathway of X-rays commonly induces severe side effects. Furthermore, the hypoxic environment around solid tumor tissue can result in significant radiation resistance. In a hypoxic environment, cancer cells can produce more glutathione (GSH, an intracellular antioxidant molecule), which helps lessen DNA damage and fragmentation. In addition, several hypoxia-inducible factors (HIFs) have been found to be related to radioresistance [2,3,4,5]. The hypoxia-associated radioresistance is thought to be crucial in clinical treatments for solid tumors of pancreatic [6], breast [2], and cervical [7] cancers.

The first strategy to reduce the radioresistance of cancer cells is reverse hypoxia. Recently, there have been some studies using hypoxia-inducible factor 1-alpha (HIF-1α) inhibitors in the treatment of hypoxic-associated resistant tumors. Yeo et al. used YC-1 (3-(5′-hydroxymethyl-2′-furyl)-1-benzimidazole) (an HIF-1 inhibitor) to repress angiogenesis and halt tumor progression [8]. Shin et al.demonstrated that YC-1 effectively inhibits tumor invasion and metastasis [9]. However, M.R. Lee et al. thought that the combination of YC-1 and radiotherapy is questionable [10], and some studies point out that excessive inhibition of HIF-1α may induce strong side effects.

Reoxygenation is another method for reducing the radioresistance caused by hypoxia. Reoxygenation, following its name, is to boost the oxygen level in tumor tissue through oxygen-containing or oxygen-producing substances. Increased intracellular oxygen concentrations can cause the degradation of HIF-1α. Clarke et al. used substances of reoxygenation as adjuvant radiation therapy and demonstrated increased lifespan in experimental animals [11]. Among oxygen-containing chemicals, perfluorocarbon (PFC, C_x_F_y_), a synthesized compound containing only fluorine and carbon, is highly capable of carrying oxygen to enhance the effect of photodynamic and photothermal therapy [12]. G. Song et al. used PFC with nanoparticles to deliver oxygen to targeted tissues for radiation therapy [13].

Another strategy for arresting radioresistance is to locally increase the radiation dosage near cancer cells via so-called radiation dose enhancers. The most common radio-enhancers are high atomic number (high-Z) metal nanoparticles such as gadolinium, ytterbium, hafnium, tantalum, tungsten, rhenium, or gold, which can easily scatter X-ray photons or emit electrons via photon excitation, Compton, or Auger effects, and even fluorescent photons [14]. In turn, these particles can trigger the production of reactive oxygen species in cells and induce DNA or organelle damage.

In this study, we adopt the second strategy by designing a nanodroplet with oxygen-carrying and radiation enhancement functions. This approach is practically suitable for the development of drug delivery from bench to bedside as the in vitro and in vivo tests can be successive and straight. The nanodroplet consists of carboxymethyl hexanoyl chitosan (CHC) as the main body, beaded with perfluorohexane (PFH) and iron oxide (superparamagnetic iron oxide, SPIO). The multifunctionality is realized through the various individual compositions in the following manner: CHC can encapsulate both hydrophobic and hydrophilic drugs, PFH effectively carries oxygen, and SPIO helps generate reactive oxygen species (ROS) through intracellular Fenton reactions. To verify the effectiveness of synthesized nanodroplets, both in vitro and in vivo experiments were carried out. In vitro experiments include the breast and cervical cancer cell models with a relatively high degree of hypoxia, while in vivo tests involve highly radioresistant breast cancer models.

## 2. Materials and Methods

### 2.1. Synthesis of SPIO@PFH-CHC Nanodroplets

CHC was synthesized using N, O-carboxymethyl chitosan (NOCC) as a precursor [14,15]. The NOCC compositions (2 g) were dissolved in distilled water (50 mL) and stirred for 24 h. The resulting solutions were mixed with methanol (50 mL, Sigma-Aldrich, St. Louis, MO, USA), following which hexanoyl anhydride (Sigma-Aldrich, MO, USA) was added at a concentration of 30 mmol. When the reaction finished, the reaction mixture was dialyzed (MW cutoff: 14,000 Da, SpectraPor^®^, Sigma-Aldrich, MO, USA) into distilled water (1 L), which was changed every 3–6 h for 2 days. After the sample was dried, CHC was obtained.

Lipophilic SPIO nanoparticles were prepared by following a method developed by Park J. et al. [16]. Briefly, 2 mmol of Fe(acac)_3_ (Sigma-Aldrich, MO, USA), 10 mmol of 1,2-hexadecanediol (Acros Organics, Geel, Belgium), 6 mmol of oleic acid (Sigma-Aldrich, Burlington, MA, USA), and 6 mmol of oleylamine (Acros Organics, Geel, Belgium) were dissolved in 20 mL of diphenyl ether (Sigma-Aldrich, Burlington, MA, USA) and refluxed at 100 °C for 30 min under a nitrogen flow. Next, the mixture was heated to 200 °C for 1 h and then heated at 265 °C for 30 min. After centrifugation and washing, the SPIO nanoparticles were collected and redispersed into ethanol.

The procedure for preparing SPIO@PFH-CHC nanodroplets was as follows: We mixed 1 mL of SPIO nanoparticles/hexane (Matrix Scientific, Elgin, SC, USA) suspension (at a concentration of 75 mg/mL) with perfluorohexane (PFH, Matrix Scientific, SC, USA), 11 μL Nile red (Sigma-Aldrich, Burlington, MA, USA)/acetone (AENCORE CHEMICAL, Whitehorse Road, Box Hill, VIC, Australia) (1.5 mg/mL), and 16 mL of CHC aqueous solution. Then the samples were sonicated in an ice bath for 10 min, followed by centrifugation and redispersion at 4 °C. Uncoated Nile red was removed by dialysis using a dialysis membrane (1000 Da) for 24 h.

### 2.2. Characterization of SPIO@PFH-CHC Nanodroplets

The drug vehicles’ mean diameter and surface zeta potential were measured using dynamic light scattering (DLS, ZS90, Malvern, Worcestershire, UK).

The morphology and size of the prepared nanoparticles were examined by TEM (JEM-2000EXII, Tokyo, Japan) and HR-TEM (JEM-1400 Plus, Tokyo, Japan) at 200 keV with energy dispersive spectroscopy (EDS, Oxford Instruments, High Wycombe, UK) for elemental composition.

X-ray photoelectron spectra (XPS, VG Scientific ESCALAB 250, Thermo Fisher Scientific, Waltham, MA, USA) were employed for analyzing the chemical binding energy spectra of SPIO@PFH-CHC nanodroplets.

### 2.3. Oxygen Loading and Release of SPIO@PFH-CHC Nanodroplets

For oxygen loading, SPIO@PFH-CHC nanodroplets (oxygen-loaded SPIO@PFH-CHC, SPIO@PFH-CHC@O_2_) were added in a 50 mL sample tube and were loaded with oxygen (O_2_ flow rate = 200 cc/min) in an aseptic biosafety cabinet for 5 min [17]. The oxygen-saturated SPIO@PFH-CHC nanodroplet is named oxygen-loaded SPIO@PFH-CHC nanodroplet. To measure the oxygen-carrying ability, 1 mL of oxygen-loaded SPIO@PFH-CHC nanodroplets was added to 3 mL of phosphate-buffered saline (PBS, Thermo Fisher Scientific, MA, USA) in a 50 mL sample tube. Following that, oxygen concentration (n = 3) was measured using a dissolved oxygen meter (DO200, Clean, Shanghai, China). To measure the oxygen release of SPIO@PFH-CHC nanodroplets, the oxygen concentration of the samples (n = 3) were measured by dissolved oxygen meter (DO200, Clean, Shanghai, China).

### 2.4. Extracellular Fenton Reaction Assay by 3,3′,5,5′-Tetramethylbenzidine (TMB)

The extracellular Fenton reaction assay was carried out in a 2 mL tube with different PBSs (pH 7.4, 6.8, 5.0). These PBSs simulated the environments of normal tissues/blood (pH 7.4), tumor tissues (pH 6.8), and lysosomes (pH 5.0), which were prepared by different ratios of disodium hydrogen phosphate and citric acid [18,19], and then 50 μL of oxygen-loaded SPIO@PFH-CHC nanodroplets, 3,3′,5,5′-tetramethylbenzidine (TMB, Acros Organics, Geel, Belgium), and H_2_O_2_ (Sigma-Aldrich, Burlington, MA, USA) were added for good mixing and incubation at 37 °C for 2 h. After incubation, absorption spectra of TMB (absorption: 652 nm) were acquired by a multimode microplate reader (200Pro, TECAN, Männedorf, Switzerland).

The use of a chemical luminescence assay to assess ROS production via H_2_O_2_ reaction with luminol is not a direct quantitative measure as the optical emission from ROS is a collective term to correlate with the amount of ROS. However, since most ROS have short lifetimes, large fluctuations are inevitable even with other tools such as liquid-phase spectrometry, especially in the intracellular environment. Thus, we consider that the commercially available TMB assay is practically adequate to quantify extracellular Fenton reactions [20,21,22,23].

### 2.5. In Vitro Experiments

#### 2.5.1. Reoxygenation Effect and HIF-1α Degradation by Oxygen-Loaded SPIO@PFH-CHC Nanodroplets

To evaluate the reoxygenation effect and HIF-1α degradation of the oxygen-loaded SPIO@PFH-CHC nanodroplets, the immunocytochemistry (ICC) method was used to detect HIF-1α, which is degraded by prolyl hydroxylase (PHD) in normoxic culture. Typically, HeLa cervical cancer cells and 4T1 murine breast cancer cells were seeded in 12-well tissue culture plate with coverslip at a density of 2 × 10^5^ cells per well in a culture medium. After overnight incubation, the culture medium was replaced with oxygen-loaded SPIO@PFH-CHC nanodroplets, and cultures were incubated for 6 h. After the incubation, the medium was removed, and the cells were washed with PBS and fixed with 4% paraformaldehyde (Acros Organics, Geel, Belgium) for 10 min. Cells were permeabilized with 0.1% Triton X-100 (Alfa Aesar, Ward Hill, MA, USA) for 15 min. After permeabilization, the samples were blocked with bovine serum albumin (BSA, Sigma-Aldrich, Burlington, MA, USA) blocking buffer for 1 h. Following blocking, the samples were incubated with HIF-1α antibody (X500, GTX127309, Genetex, Irvine, CA, USA) at 4 °C for overnight. And then, the samples were incubated with goat anti-rabbit IgG antibody (X500, DyLight594, GTX213110-05, Genetex, CA, USA) for 1 h. After immunostaining, the cells were stained with Alexa Fluor™ 488 phalloidin (Thermo Fisher Scientific, MA, USA) for 30 min. Finally, the cells were examined using an inverted laser scanning confocal microscope with AiryScan (LSM880, Zeiss, Jena, Germany).

#### 2.5.2. Cellular Uptake of Nile Red and Oxygen-Loaded SPIO@PFH-CHC Nanodroplets

To investigate the intracellular uptake of an oxygen-loaded SPIO@PFH-CHC nanodroplet, Nile red (1.5 mg/mL) was encapsulated in the oxygen-loaded SPIO@PFH-CHC nanodroplet. Typically, L929 normal fibroblasts, HeLa cervical cancer cells, and 4T1 murine breast cancer cells were seeded in a 12-well tissue culture plate with a coverslip at a density of 2 × 10^5^ cells per well in a culture medium. After overnight incubation, the culture medium was replaced with Nile red-loading oxygen-loaded SPIO@PFH-CHC nanodroplets, and cultures were incubated for 0, 2, 4, and 6 h. After the incubation, the medium was removed, and the cells were washed with PBS and fixed with 4% paraformaldehyde for 10 min. After washing twice with PBS, the cells were stained with Alexa Fluor™ 488 phalloidin (Thermo Fisher Scientific, MA, USA) for 30 min, and the coverslips were mounted with a Fluoroshield mounting medium with 4′,6-diamidino-2-phenylindole (DAPI) (Abcam, Cambridge, UK). Finally, the cells were examined using an inverted laser scanning confocal microscope with AiryScan (LSM880, Zeiss, Jena, Germany).

#### 2.5.3. Intracellular Hydrogen Peroxide Degradation by Oxygen-Loaded SPIO@PFH-CHC Nanodroplets

For the detection of the intracellular Fenton reaction, the degradation of the intracellular H_2_O_2_ assay was used. When the Fenton reaction is active inside of the cell, the process must cost out the intracellular H_2_O_2_; for this reason, we can depend on the degradation of intracellular H_2_O_2_ to define the activity of the intracellular Fenton reaction. HeLa cervical cancer cells and 4T1 murine breast cancer cells were seeded in a 6-well tissue culture plate at a density of 5 × 10^5^ cells per well in a culture medium. After overnight incubation, the culture medium was replaced with oxygen-loaded SPIO@PFH-CHC nanodroplets (various concentrations = 1600 μg/mL for HeLa cells; 800 μg/mL for 4T1 cells) and cultures were incubated for 6 h. After incubation, the cell culture was resuspended in 100 μL culture medium with the CellROX™ Deep Red Reagent, followed by incubation for 30 min. Finally, the cells were examined using flow cytometry (CytoFLEX, Beckman, Brea, CA, USA) in the allophycocyanin (APC, 638 nm laser, 660 nm/20 nm filer) channel.

#### 2.5.4. Influence of Cell Cycle by Oxygen-Loaded SPIO@PFH-CHC Nanodroplets

In vitro X-ray irradiation for cancer cells was generated by a linear accelerator (Clinac 2100c, Varian Inc., Palo Alto, CA, USA), set to 6 MV and a 150 rad/min dose rate. All samples were placed 100 cm from the source to ensure a uniform dose distribution. The dosimetry was verified using an equivalent water-filled phantom to guarantee the desired dose delivery with minimum errors.

HeLa cervical cancer cells and 4T1 murine breast cancer cells were seeded in the 6-well culture plate at a concentration of 5 × 10^5^ cells per well in the culture medium. After overnight incubation, the culture medium was replaced and incubated with a serum-free culture medium (control group), SPIO@PFH-CHC nanodroplets (SPIO@PFH-CHC group), an oxygen-loaded culture medium (reoxygenation group), and oxygen-loaded SPIO@PFH-CHC nanodroplets (SPIO@PFH-CHC@O_2_ group) (various concentrations = 1600 μg/mL for HeLa cells; 800 μg/mL for 4T1 cells) for 6 h. After incubation, the cultures were subjected to X-ray irradiation (6 MV, 2 Gy) or not, and then incubated for 24 h.

The DNA fragmentation of cells was determined using propidium iodide (PI, Thermo Fisher Scientific, MA, USA)). First, the cell cultures were washed twice with ice-cold PBS and fixed with ice-cold 70% ethanol overnight. After the fixation, cultured cells were rehydrated and stained with a staining buffer (Ribonuclease (RNase, Genetex, CA, USA): 0.2 mg/mL; PI: 20 μg/mL) for 30 min. Finally, the cells were examined using flow cytometry (CytoFLEX, Beckman, CA, USA) in the phycoerythrin (PE, 488 nm laser, 585 nm/42 nm filter) channel.

#### 2.5.5. In Vitro Cytotoxicity of SPIO@PFH-CHC Nanodroplets

L929 normal fibroblasts were seeded in a 24-well tissue culture plate at a density of 5 × 10^4^ cells per well in a culture medium. After overnight incubation, the culture medium was replaced with a different concentration of SPIO@PFH-CHC nanodroplets, and the culture was incubated for 2 h. After incubation, the cell cultures were subjected to X-ray irradiation (6 MV, 2 Gy) or not, and then incubated for 24, 48, and 72 h. The cell viability was determined using the PrestoBlue™ Cell Viability Reagent (Thermo Fisher Scientific, MA, USA): 300 μL of 5% PrestoBlue Solution/without a serum culture medium was added into each well and incubated for 15 min. Finally, the fluorescence spectra of PrestoBlue™ (excitation/emission: 560 nm/590 nm) were acquired by a multimode microplate reader (200Pro, TECAN, Männedorf, Switzerland). The untreated cells as control group, and the results of the biocompatibility were derived (n = 6).

HeLa cervical cancer cells and 4T1 murine breast cancer cells were seeded in a 12-well tissue culture plate at a density of 1 × 10^5^ cells per well in a culture medium. After overnight incubation, the culture medium was replaced and incubated with a serum-free culture medium (control group), SPIO@PFH-CHC nanodroplets (SPIO@PFH-CHC group), an oxygen-loaded culture medium (reoxygenation group), and oxygen-loaded SPIO@PFH-CHC nanodroplets (SPIO@PFH-CHC@O_2_ group) (various concentrations = 1600 μg/mL for HeLa cells; 800 μg/mL for 4T1 cells) for 6 h. After incubation, the cultures were subjected to X-ray irradiation (6 MV, 2 Gy) or not, and then incubated for 24 to 48 h. The cell viability was determined using the PrestoBlue™ Cell Viability Reagent (Thermo Fisher Scientific, MA, USA): 200 μL of 5% PrestoBlue Solution/without a serum culture medium was added into each well and incubated for 15 min. Finally, the fluorescence spectra of PrestoBlue™ (excitation/emission: 560 nm/590 nm) were acquired by a multimode microplate reader (200Pro, TECAN, Männedorf, Switzerland). The control group was untreated cells (n = 6).

#### 2.5.6. Clonogenic Assay for the Proliferation of Cells Under Oxygen-Loaded SPIO@PFH-CHC Nanodroplets

HeLa cervical cancer cells and 4T1 murine breast cancer cells were seeded in the 6-well tissue culture plate at a density of 5 × 10^5^ cells per well in the culture medium. After overnight incubation, the culture medium was replaced and incubated with a serum-free culture medium (control group), SPIO@PFH-CHC nanodroplets (SPIO@PFH-CHC group), an oxygen-loaded culture medium (reoxygenation group), and oxygen-loaded SPIO@PFH-CHC nanodroplets (SPIO@PFH-CHC@O_2_ group) (various concentrations = 1600 μg/mL for HeLa cells; 800 μg/mL for 4T1 cells) for 6 h. After incubation, the cultures were subjected to X-ray irradiation (6 MV, 2 Gy) or not. Following the irradiation, the cell culture would re-seed to a new 6-well tissue culture plate at a density of 200 cells per well for HeLa cells and 100 cells per well for 4T1 cells. After 120 h incubation, the culture medium was removed by PBS twice. Then cells were fixed and stained with a staining buffer of 6.0% glutaraldehyde and 0.5% crystal violet (Acros Organics, Geel, Belgium) for 60 min. The excessive staining buffer was washed out again by PBS.

The 6-well tissue culture plates were photographed for the colonies counting under a white paper background. The images were preprocessed by ImageJ (version 1.51t) by enhancing contrast at first. Then colonies were counted by ImageJ and cross-verified by manually counting.

### 2.6. In Vivo Experiment

Five-week-old nude female mice with body weights of around 18 ± 2 g were purchased from the National Laboratory Animal Center. All animals used in our experiments were treated and housed according to a protocol approved by the Institutional Animal Care and Use Committee of National Yang Ming Chiao Tung University (NYCU-IACUC 1080316). Then 4T1 cells (2 × 10^6^ cells) suspended in 100 μL of PBS were injected into the right back of each nude mouse.

#### 2.6.1. Anti-Tumoral Effect by Oxygen-Loaded SPIO@PFH-CHC Nanodroplets

The tumor-bearing mice were subjected to treatments when the tumor volume grew to around 100 mm^3^. The tumor volume was estimated using the following equation:

(1) $$\mathrm{Tumor}\;\mathrm{volume}\;\left({\mathrm{mm}}^{3}\right)=\frac{4\pi }{3}\times abc\approx \frac{\mathrm{length}\times {\mathrm{width}}^{2}}{2}$$

where *a*, *b*, and *c* are the lengths of the semiaxes. The approximation assumes one major axis as the length, two equal semiaxes as the width, and *π* ≈ 3. Such an approximation makes it easier to estimate the volume of a tumor measured by a caliper.

The in vivo anti-tumor effect was studied among different groups, and each group has three mice: Group A: control; Group B: radiation therapy; Group C: oxygen-loaded SPIO@PFH-CHC nanodroplets; Group D: SPIO@PFH-CHC nanodroplets + radiation therapy; and Group E: oxygen-loaded SPIO@PFH-CHC nanodroplets + radiation therapy. The SPIO@PFH-CHC nanodroplets and oxygen-loaded SPIO@PFH-CHC nanodroplets were intratumorally injected into the tumor center, and the radiation therapy was subjected to X-ray (6 MV, 2 Gy) for 6 h after the injection of either SPIO@PFH-CHC or oxygen-loaded SPIO@PFH-CHC nanodroplets. Before X-ray irradiation, the tumor-bearing mice were anesthetized using Ketamine/Xylazine (100 mg/kg: 10 mg/kg, intraperitoneal injection) to ensure immobility and minimum stress on mice. Once the anesthesia was confirmed, the mice were placed in special poly (methyl methacrylate) fixators designed to securely immobilize the animals. X-ray irradiation was performed with a linear accelerator (Clinac 2100c, Varian Inc., CA, USA) set to 6 MV and a 150 rad/min dose rate. The mice were placed 100 cm from the source for a uniform dose distribution. The treatment was conducted for five cycles in 15 days. The volume of the tumor was measured twice every week.

#### 2.6.2. In Vivo Biocompatibility for Oxygen-Loaded SPIO@PFH-CHC Nanodroplets

In vivo biocompatibility is based on mice body weight change to analyze the side effects of oxygen-loaded SPIO@PFH-CHC nanodroplets. The body weights of the mice were measured two times each week.

### 2.7. Statistical Analysis

The statistics of experimental results were analyzed using the GraphPad Prism software (Prism 9, GraphPad Software, Boston, MA, USA). All data in this study were presented as means ± standard deviation. Two-way analysis of variance (two-way ANOVA) was used to make comparisons among different groups of data. A *p*-value of 0.05 was considered to be statistically significant.

## 3. Results and Discussion

### 3.1. Characterization and Oxygen Carrying of SPIO@PFH-CHC Nanodroplets

**Figure 1:**
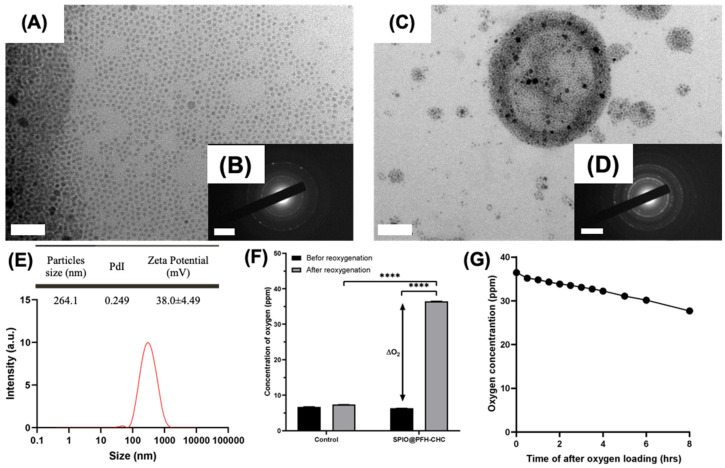
The characterization and functions of SPIO NPs and SPIO@PFH-CHC nanodroplets. (**A**) TEM image of SPIO NPs, (**B**) electron diffraction of SPIO NPs, (**C**) TEM image of SPIO@PFH-CHC nanodroplets, and (**D**) electron diffraction of SPIO@PFH-CHC nanodroplets. The scale bar in (**A**) is 30 nm, in (**C**) is 50 nm, and in (**B**,**D**) is 2 1/nm. (**E**) The size distribution of SPIO@PFH-CHC nanodroplets is measured by DLS. (**F**) The concentrations of oxygen carrying in SPIO@PFH-CHC nanodroplets before and after (5 min) reoxygenation (n = 3; mean ± std. dev.; **** *p* < 0.0001). (**G**) The release of oxygen by SPIO@PFH-CHC nanodroplets after oxygen loading (n = 3).

Figure 1A–D show the TEM and electron diffraction of SPIO nanoparticles and SPIO@PFH-CHC nanodroplets. The hydrophobic SPIO nanoparticles synthesized by thermal degradation have a diameter of about 4 nm, a uniform shape, and good dispersion. These SPIO particles were successfully associated with SPIO@PFH-CHC nanodroplets around 264.1 nm in diameter. Figure 1E further shows that the polydispersity index (PdI) is 0.249 and the surface zeta potential is 38.0 ± 4.49 mV. Overall, SPIO@PFH-CHC nanodroplets are spherical with a core-shell structure formed with the hydrophobic groups in CHC molecules and hydrophobic SPIO nanoparticles as the outer shell and the hydrophobic PFH as the inner core. The oxygen-carrying capacity of SPIO@PFH-CHC nanodroplets is shown in Figure 1F, where the oxygen concentration is increased by 30.13 ppm at 5 min after oxygen loading, in contrast to that in the control group (without SPIO@PFH-CHC nanodroplets). This result directly shows the function of oxygen carrying by PFH. In the oxygen release test (Figure 1G), the oxygen-loaded SPIO@PFH-CHC nanodroplets exhibited a slow release. Less than 25% of the loaded oxygen escaped after an 8 h measure.

### 3.2. The Extracellular Fenton Reaction Assay

**Figure 2:**
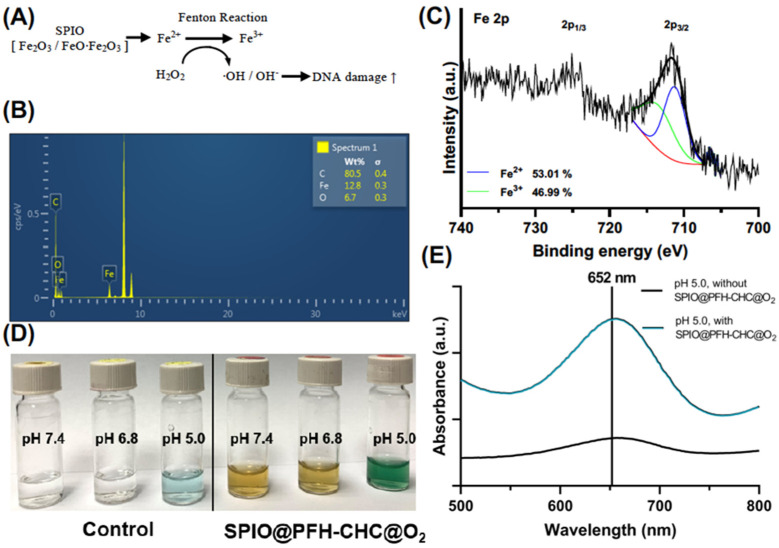
The extracellular Fenton reaction assay. (**A**) The process of the chemodynamic therapy (CDT) and intracellular Fenton-like reaction. (**B**) EDS of SPIO@PFH-CHC nanodroplets. (**C**) XPS of Fe 2p in the SPIO@PFH-CHC nanodroplets. (**D**) Right: the color change of tetramethyl benzidine (TMB + H_2_O_2_) by oxygen-loaded SPIO@PFH-CHC nanodroplets via Fenton reaction at different pH levels. Left: the control group without nanodroplets (TMB + H_2_O_2_). All samples were assessed 120 min after incubation. (**E**) The optical absorbance at 652 nm for samples in (**D**) at pH 5.0.

The Fenton process shown in Figure 2A requires hydrogen peroxide H_2_O_2_ and Fe(II) to oxidize organic chemicals. The process starts with Fe(II) oxidized by H_2_O_2_ to Fe(III), forming a hydroxyl radical HO^•^ and a hydroxide ion OH^−^. Then Fe(III) is reduced back to Fe(II) by another H_2_O_2_, forming a hydroperoxyl radical HOO^•^ and a proton (H^+^). The net reaction is a *disproportionation* of H_2_O_2_ to create HO^•^ and HOO^•^ with H_2_O as a byproduct. Fe(II) serves as a catalyst in the whole process.

EDS in Figure 2B confirmed the presence of Fe in the SPIO@PFH-CHC nanodroplet. The oxidation state of Fe in SPIO@PFH-CHC nanodroplets was further examined by XPS. As shown in Figure 2C, peaks of Fe 2p_1/3_ and Fe 2p_3/2_ were identified at 725.2 eV and 711.7 eV, respectively. The Fe 2p_3/2_ peak can be further split into two peaks at 711.2 eV and 713.5 eV corresponding to Fe^2+^ and Fe^3+^ [24,25,26]. The proportions of Fe^2+^ and Fe^3+^ were found to be 53.01% and 46.99% according to the area by numerical fitting.

To further investigate the hydroxyl radical HO^•^ produced by the catalytic decomposition of H_2_O_2_ in the Fenton process, we used the tetramethylbenzidine (TMB) assay (TMB + H_2_O_2_) to assess the effect of oxygen-loaded SPIO@PFH-CHC nanodroplets in PBS solutions at different pH levels. Note that the color of TMB is changed from transparent to blue-green according to the degrees of oxidation. The optical absorbance of this color change centers at ~650 nm.

Figure 2D demonstrates the color change of TMB at pH 5.0, 6.8, and 7.4 (pH 7.4: normal tissue environment; pH 6.8: cancerous tissue environment; pH 5.0: intra-lysosomal environment). The original colors of TMB at different pH values are shown in the control group (no SPIO@PFH-CHC nanodroplets). When the SPIO@PFH-CHC nanodroplets are added to PBS, the oxidation of TMB causes the color change from light brown (pH 7.4) to dark blue-green (pH 5.0). The optical absorbance at 652 nm for samples at pH 5.0 is presented in Figure 2E for a quantitative comparison. It indicates that oxygen-loaded SPIO@PFH-CHC nanodroplets favor an acidic environment to more effectively oxidize TMB via the hydroxyl radical HO^•^ produced by Fenton reactions.

As an annotation for the experimental results in Figure 2, the Fe^2+^ in SPIO@PFH-CHC nanodroplets can interact with hydrogen peroxide (H_2_O_2_) in lysosomes and decompose hydrogen peroxide in an acidic environment through the Fenton process. The generated hydroxyl radicals (HO^•^) would have an active role in the oxidation of other molecules in cells.

### 3.3. Effects of In Virto Reoxygenation by Oxygen-Loaded SPIO@PFH-CHC Nanodroplets

**Figure 3:**
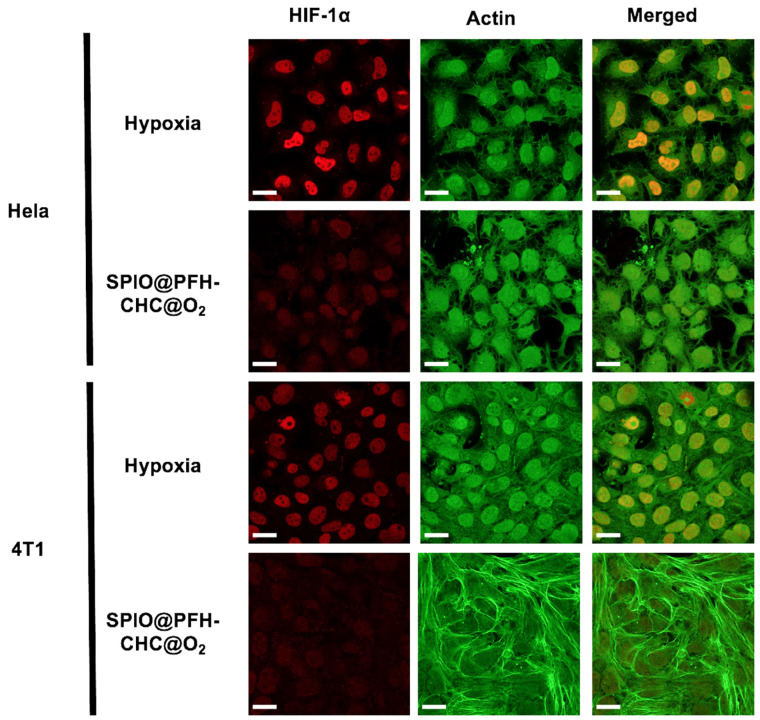
The degradation of hypoxia-inducible factor 1-alpha (HIF-1α) by oxygen-loaded SPIO@PFH-CHC nanodroplets. The HeLa cervical cancer cells and 4T1 breast cancer cells were incubated with oxygen-loaded SPIO@PFH-CHC nanodroplets (800 μg/mL) for 6 h. The anti-HIF-1α (red) and cell filamentous actin (green) were stained by Thermo Fisher Alexa Fluor 488 phalloidin. The scale bar is 20 μm.

Figure 3 presents the immune-stained HeLa cervical cancer cells and 4T1 mouse breast cancer cells cultured with oxygen-loaded SPIO@PFH-CHC nanodroplets (reoxygenation state) or without (hypoxia state) for 6 h. The fluorescent color of anti-HIF-1α is red, and that of cell filamentous actin is green. Overlapping both stained images shown in the third column would allow us to inspect the presence of HIF-1α inside cells. These images clearly indicate that the fluorescent intensity of anti-HIF-1α diminishes when oxygen-loaded SPIO@PFH-CHC nanodroplets are present in the culture media. In contrast to cases without SPIO@PFH-CHC nanodroplets, the red anti-HIF-1α is expressed in most cells.

Figure 3 supports the idea that reoxygenation by the oxygen-loaded SPIO@PFH-CHC nanodroplets can help degrade HIF-1α. This is an important feature of nanodroplets in reducing the radioresistance of cancer cells when exposed to X-ray irradiation.

### 3.4. The Cellular Uptake and Intercellular Fenton Reaction Assay

**Figure 4:**
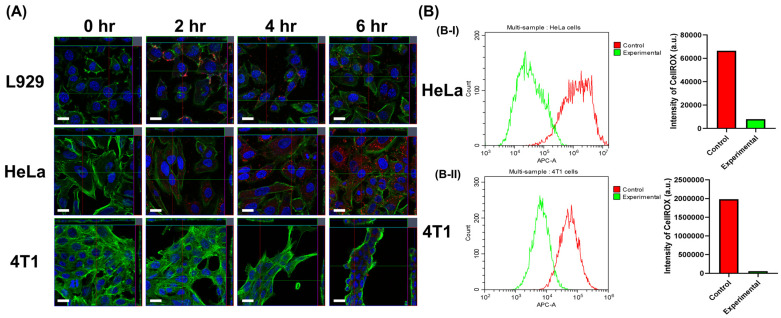
The cellular uptake and intercellular Fenton reaction assay. (**A**) The confocal microscopic images of cellular uptake (L929, HeLa, and 4T1) of Nile red and oxygen-loaded SPIO@PFH-CHC nanodroplets (800 μg/mL) at 0, 2, 4, and 6 h after incubation. For each image, the color blue is cell-nuclei-stained by DAPI, red is Nile red and oxygen-loaded SPIO@PFH-CHC nanodroplets, and green is cell filamentous actin stained by Alexa Fluor 488 phalloidin. The scale bar is 20 μm. (**B**) The decomposition of H_2_O_2_ by oxygen-loaded SPIO@PFH-CHC nanodroplets via intracellular Fenton reaction for HeLa cervical cancer cells ((**B-I**), 1600 μg/mL) and 4T1 breast cancer cells ((**B-II**), 800 μg/mL) at 6 h. ROS was measured by the APC channel of flow cytometry. The control group is cells cultured without SPIO@PFH-CHC nanodroplets.

The uptake by cancer cells of oxygen-loaded SPIO@PFH-CHC nanodroplets is shown in Figure 4, where the fluorescent color of red is Nile red and oxygen-loaded SPIO@PFH-CHC nanodroplets, blue is cell nuclei stained by DAPI, and green is cell filamentous actins stained by Alexa Fluor 488 phalloidin. The images were taken at 0, 2, 4, and 6 h after seeding. L929 normal fibroblasts were included as the control group to compare with HeLa cervical cancer cells and 4T1 mouse breast cancer cells.

Figure 4A shows that L929 would not ingest SPIO@PFH-CHC nanodroplets as red fluorescence stayed on the cell membrane surface shown in the 3D images. Both the Hela cervical cancer cell and the 4T1 mouse breast cancer cell started to take in a small number of SPIO@PFH-CHC nanodroplets after 2 and 4 h of culture, indicated by the red fluorescence spots in the cytoplasm. The intensity of red fluorescence reaches a maximum at 6 h of culture. That is to say, Hela cells and 4T1 cells began to ingest more nanodroplets after 4 h and reached the highest point at 6 h.

As mentioned previously, the decomposition of H_2_O_2_ by oxygen-loaded SPIO@PFH-CHC nanodroplets can be realized via intracellular Fenton reaction. Figure 4B demonstrates the concentration of stained ROS inside HeLa cervical cancer cells (B-I, 1600 μg/mL) and 4T1 breast cancer cells (B-II, 800 μg/mL) at 6 h after culture. Compared with the control groups, which are cells cultured without the oxygen-loaded SPIO@PFH-CHC nanodroplets in the media, the much-decreased ROS counts by flow cytometry indicate that the uptake of SPIO@PFH-CHC nanodroplets significantly increased both cancer cells at 6 h after culture, but also induced an extensive decomposition of ROS inside the cells.

It is worth mentioning that the intracellular decomposition of ROS implies the function of Fe^2+^ from oxygen-loaded SPIO@PFH-CHC nanodroplets in Fenton reactions. This decomposition could lower the pH value inside cells because of the production of hydroxyl radicals (HO^•^) and hydroxide ions (OH^−^). Both are harmful to the DNA.

Moreover, the interval between intratumoral injection of SPIO@PFH-CHC@O_2_ nanodroplets and radiotherapy was set to 6 h. This time was determined based on the in vitro results that the cellular uptake of oxygen-loaded nanodroplets peaked approximately 6 h after incubation (Figure 4A). This time is coincident with the measurement of oxygen release and the degradation of HIF-1α, an indication of maximum oxygen availability around that time frame. In other words, a 6 h interval between injection and radiotherapy could ensure enhanced oxygenation levels in the cellular microenvironment prior to radiotherapy.

### 3.5. Effects of Radiotherapy and Oxygen-Loaded SPIO@PFH-CHC Nanodroplets on Cell Cycle and DNA Fragmentation

**Figure 5:**
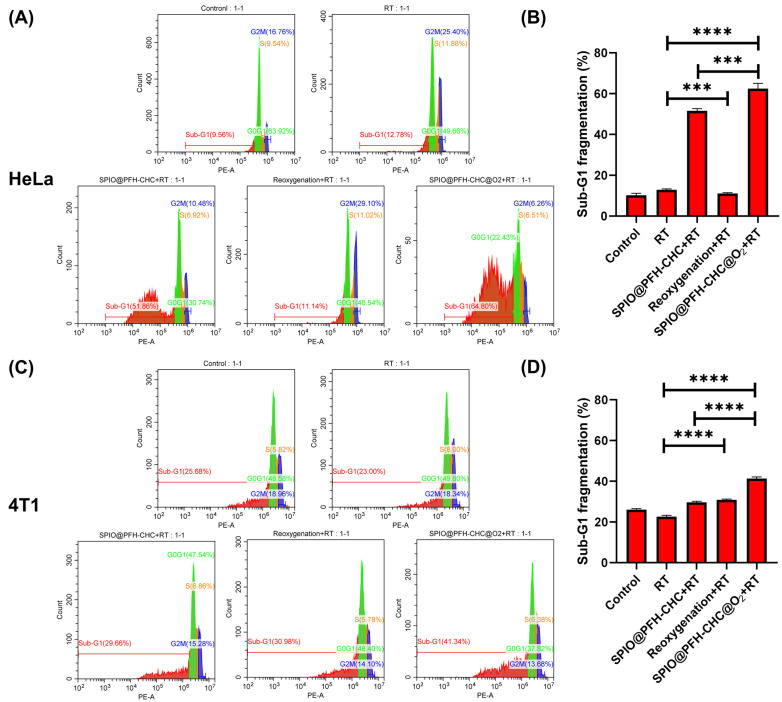
The cell cycles and DNA fragmentation of the HeLa cells and 4T1 cells with different treatments (control: no treatment; RT: radiotherapy only; SPIO@PFH-CHC nanodroplets + RT: SPIO@PFH-CHC nanodroplets and radiotherapy; reoxygenation + RT: oxygen-loaded culture medium and radiotherapy; SPIO@PFH-CHC@O_2_ nanodroplets + RT: oxygen-loaded SPIO@PFH-CHC nanodroplets and radiotherapy). The cell cycle under cytometry is divided into the G0G1 (green), S (orange), and G2M (blue) phases. (**A**) DNA fragmentation in different phases and (**B**) sub-G1 phase in HeLa cervical cancer cells. (**C**) DNA fragmentation in different phases and (**D**) sub-G1 phase in 4T1 breast cancer cells (n = 3; mean ± std. dev.; *** = *p* < 0.0005; **** *p* < 0.0001).

The effects of five different treatments on both cancer cells are shown in Figure 5 for the cell cycle analysis and DNA fragmentation measured by cell cytometry. The combined treatments are no treatment (control), radiotherapy only (RT), SPIO@PFH-CHC nanodroplets and radiotherapy (SPIO@PFH-CHC + RT), oxygen-loaded culture medium and radiotherapy (reoxygenation + RT), and oxygen-loaded SPIO@PFH-CHC nanodroplets and radiotherapy (SPIO@PFH-CHC@O_2_ + RT). The dose of the radiotherapy is X-ray irradiation (6 MV, 2 Gy).

In the cycle analysis, since the propidium iodide (PI) stain intercalates between two nitrogenous base pairs of DNA, the fluorescent intensity of propidium iodide is proportional to the molecule weight of DNA. When a double-strand DNA breaks, its molecular weight decreases to less than that of the DNA in the G1 phase, and so is the intensity of PI fluorescence.

The cell cycle analysis and DNA fragmentation in Figure 5A,B are for HeLa cervical cancer cells and in Figure 5C,D for 4T1 mouse breast cancer cells. The sub-G1 phase is particularly highlighted as it is a good indicator for apoptotic cells or cells that lost sufficient DNA. Note that two exceptions may be possible and not counted in sub-G1: cells enter apoptosis from either the S or G2/M phase or aneuploid cells undergo apoptosis. However, these cases are usually assumed to be marginal.

Among five treatments, the effects of SPIO@PFH-CHC + RT and SPIO@PFH-CHC@O_2_ + RT are significant for DNA fragmentation in HeLa cells. Meanwhile, SPIO@PFH-CHC + RT, reoxygenation + RT, and SPIO@PFH-CHC@O_2_ + RT are beneficial for DNA fragmentation in 4T1 cells. Comparing two cell lines, SPIO@PFH-CHC + RT and SPIO@PFH-CHC + RT are much more effective in damaging HeLa’s DNAs (51.86% and 64.80% vs. 29.66–41.34% of DNA fragmentation in the sub-G1 phase).

It may be even more instructive to look at the G2/M phase where for HeLa cells, SPIO@PFH-CHC + RT and SPIO@PFH-CHC + RT result in 10.48% and 6.26% of the cells entering. In 4T1 cells, SPIO@PFH-CHC + RT, reoxygenation + RT, and SPIO@PFH-CHC@O_2_ + RT lead to 15.25%, 14.10%, and 13.68% of the cells reaching the G2/M phase. These numbers explain the threat to the proliferation of both cells due to DNA defragmentation by combining the drug and X-ray.

Figure 5 can be viewed as a comprehensive study of the combined effects of using drugs and radiotherapy. The oxidative effects through possible Fenton reactions by oxygen-loaded SPIO@PFH-CHC nanodroplets found in Figure 3 and Figure 4 can be a synergism with the X-ray irradiation, which jointly augment the DNA damages in cancer cells. Figure 5 highlights key differences between SPIO@PFH-CHC + RT and SPIO@PFH-CHC@O_2_ + RT treatments in terms of DNA fragmentation and cell cycle effects. These findings suggest that the oxygen loading of SPIO@PFH-CHC nanodroplets significantly amplifies the oxidative stress and DNA damage induced by radiotherapy.

### 3.6. In Vitro Cytotoxicity Assay of Oxygen-Loaded SPIO@PFH-CHC Nanodroplets

**Figure 6:**
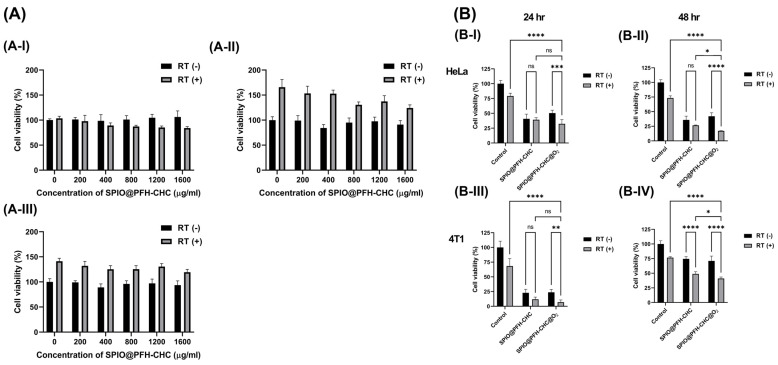
In vitro cytotoxicity assay of oxygen-loaded SPIO@PFH-CHC nanodroplets measured by the PrestoBlue cell viability assay. (**A**) L929 fibroblasts (normal cells) with (RT(+))/without (RT(−)) X-ray irradiation (6 MV, 2 Gy). (**A-I**) is 24 h, (**A-II**) is 48 h, and (**A-III**) is 72 h after irradiation. (**B**) HeLa cervical cancer cells and 4T1 breast cancer cells with different treatments. SPIO@PFH-CHC nanodroplets are 1600 μg/mL for HeLa cells and 800 μg/mL for 4T1 cells, then incubated for 24 and 48 h (n = 3; mean ± std. dev.; ns = non-significant; * = *p* < 0.05; ** = *p* < 0.01; *** = *p* < 0.0005; **** *p* < 0.0001).

Figure 6A shows the in vitro toxicity of various concentrations (0–1600 μg/mL) of SPIO@PFH-CHC nanodroplets to L929 normal fibroblasts evaluated by the PrestoBlue™ assay. (A-I) is 24 h, (A-II) is 48 h, and (A-III) is 72 h after seeding either with (RT(+)) or without (RT(−)) X-ray irradiation (6 MV, 2 Gy). The decline of cell count from samples at 72 h should be attributed to the deficit of nutrients and space because of overcrowded cells at 48 h.

Although L929 cells would not uptake SPIO@PFH-CHC nanodroplets (Figure 4A), the X-ray irradiation can ionize the culture media and SPIO@PFH-CHC nanodroplets to produce a very low quantity of oxygen ions or radicals. These ROS may help with the proliferation of L929 in 48 and 72 h after culture [27,28,29,30,31].

Further explanations are needed here. We notice that an unexpectedly higher cell viability in Figure 6(A-II) and Figure 6(A-III) was observed in the radiation therapy (RT(+)) group compared with the non-radiation therapy group (RT(−)) even in the absence of SPIO@PFH-CHC nanodroplets. This anomaly may be explained by the low levels of reactive oxygen species (ROS) generated by low-dose radiation, which may stimulate cell proliferation through redox signaling pathways such as the extracellular signal-related kinase (ERK) or the nuclear factor-*κ*B (NF-*κ*B). Other research has shown that at low concentrations, ROS can act as signaling molecules that activate cellular processes, including proliferation and stress adaptation, rather than oxidative stress-induced damages [27,28,29,30,31].

We also notice that these nanodroplets are like local oxygen centers that can lead to energy surges by X-ray absorption and cause more extensive DAN damage.

For HeLa cells, the oxygen-loaded SPIO@PFH-CHC nanodroplets have effectively suppressed the cell viability whether under O_2_ supply (Figure 6(B-I)) or without O_2_ supply (Figure 6(B-II)).

The oxygen-loaded SPIO@PFH-CHC nanodroplets have similar effects on suppressing the cell viability for 4T1 whether with (Figure 6(B-III)) or without (Figure 6(B-IV)) O_2_ supply. The hypoxia condition (Figure 6(B-IV)) is beneficial to 4T1 cells but oxygen-loaded SPIO@PFH-CHC nanodroplets still have their potency against the growth of 4T1 cells.

However, in this experiment, the evaluation of the long-term therapeutic effect of the treatment has been limited by the timeline. More extended observation periods are crucial to assess the immediate impact on cancer cell growth and DNA damage and the long-term therapeutic effect of treatments, which was evaluated by the result of the clonogenic assay.

### 3.7. The Radio- and Fenton Sensitivity of HeLa and 4T1 Cells via Clonogenic Assay

**Figure 7:**
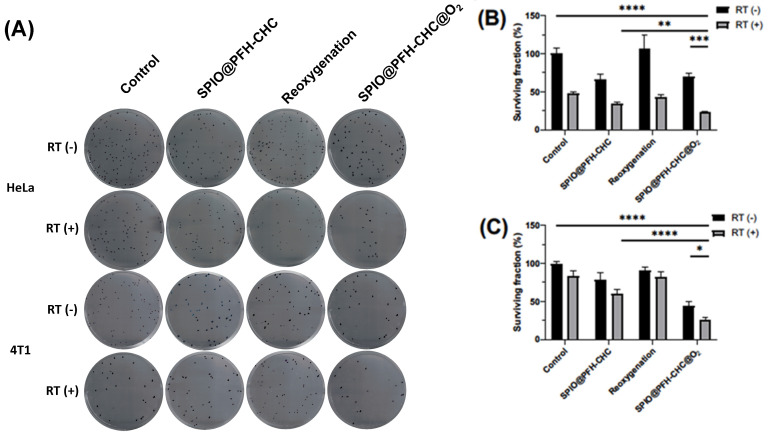
The radio- and Fenton sensitivity of HeLa cervical cancer cells and 4T1 breast cancer cells via clonogenic assay with (RT(+))/without (RT(−)) X-ray irradiation (6 MV, 2 Gy). (**A**) The representative image of the clonogenic assay for HeLa cells and 4T1 cells by different treatments. (**B**) The survival colonies in HeLa cells and (**C**) 4T1 cells. The SPIO@PFH-CHC nanodroplets are 1600 μg/mL for HeLa cells and 800 μg/mL for 4T1 cells. Cells exposed to X-ray irradiation after 24 h. The re-seed cell concentration is 200 cells/well for HeLa cells and 100 cells/well for 4T1 cells in 6-well plates (n = 3; mean ± std. dev.; * = *p* < 0.05; ** = *p* < 0.01; *** = *p* < 0.0005; **** *p* < 0.0001).

Figure 7 shows the clonogenic assay for HeLa and 4T1 cells where cells were cultured under four different conditions (control, SPIO@PFH-CHC nanodroplets, oxygen-loaded media, and oxygen-loaded SPIO@PFH-CHC nanodroplets). After 24 h culture, cells were exposed to X-ray irradiation (6 MV, 2 Gy) and re-seeded for another round of 24 h culture. The re-seeding cell concentration is 200 cells/well for HeLa cells and 100 cells/well for 4T1 cells in 6-well plates.

The reduced number of colonies in Figure 7A (all cases of RT(+)) confirms the effect of radiotherapy, similar to the observation shown in Figure 6B for cases at 24 h after culture.

Figure 7B shows the survival colonies of HeLa cells after SPIO@PFH-CHC nanodroplet treatments and X-ray irradiation. All three combined treatments are effective against HeLa cells. This result is similar to Figure 5B except in the case of reoxygenation (oxygen-loaded media).

For 4T1 cells, Figure 7C shows that the combined SPIO@PFH-CHC nanodroplet and oxygen-loaded SPIO@PFH-CHC nanodroplet treatments with X-ray irradiation are more effective. This result is in accordance with the results in Figure 6B.

Figure 7B,C demonstrate that both SPIO@PFH-CHC nanodroplet and SPIO@PFH-CHC@O_2_ nanodroplet treatments alone effectively reduce the clonogenic survival of HeLa and 4T1 cells, indicating that Fe^2+^-mediated Fenton reactions contribute to DNA damage via hydroxyl radical (HO^•^) production. However, the oxygen-loaded SPIO@PFH-CHC@O_2_ nanodroplets exhibit significantly greater efficacy in combination with radiotherapy (RT).

In fact, Figure 7B,C also demonstrate that with just SPIO@PFH-CHC nanodroplet and oxygen-loaded SPIO@PFH-CHC nanodroplet treatments alone, both cancer cells are impacted by the Fe2^+^-related Fenton process to create hydroxyl radicals HO^•^ and a hydroxide OH^−^ inside cells and cause some DNA damages.

It is found that current oxygen-loaded SPIO@PFH-CHC nanodroplets are more lethal to HeLa cells than 4T1 mouse breast cancer cells based on the results in Figure 5B and Figure 7B,C. We think that 4T1 cells may have been more resistant to the oxygen environment in this study.

### 3.8. In Vivo Experiment for Oxygen-Loaded SPIO@PFH-CHC Nanodroplets

**Figure 8:**
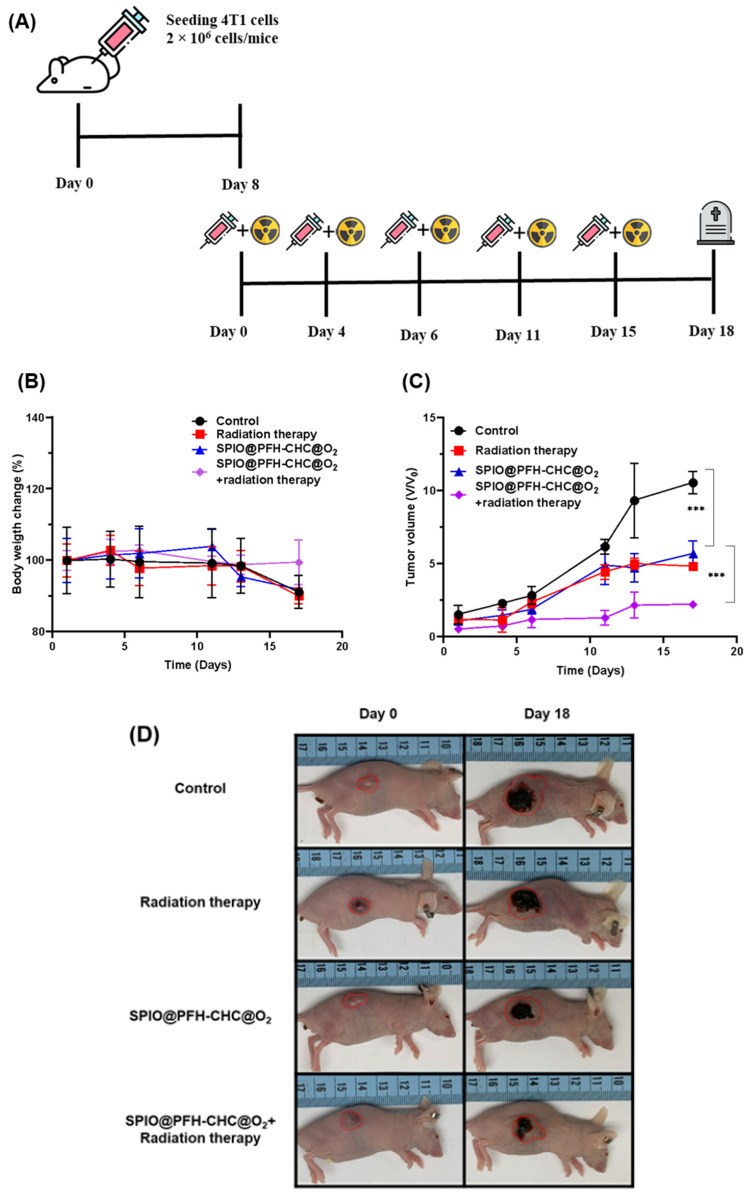
In vivo animal model for oxygen-loaded SPIO@PFH-CHC nanodroplets. (**A**) Schematic drawing of the experimental producer. (**B**) The ratios of body weights of mice after different treatments. (**C**) The ratios of tumor volumes after different treatments. Treatment group: control, radiation therapy, oxygen-loaded SPIO@PFH-CHC nanodroplets, and oxygen-loaded SPIO@PFH-CHC nanodroplets + radiation therapy. (**D**) The images of tumors with scales. The dose of X-ray radiation is (6 MV, 2 Gy), and nanodroplets are 0.2 mg/time (n = 3; mean ± std. dev.; *** = *p* < 0.0005). (**D**) The surface morphology of the tumor after different treatments.

Figure 8A shows the animal model of tumor-bearing mice treated for the in vivo anti-tumor effect. Five groups of mice were subjected to the test, and each group had three mice. These groups are A: control; B: radiation therapy only; C: oxygen-loaded SPIO@PFH-CHC nanodroplets only; D: SPIO@PFH-CHC nanodroplets and radiation therapy; and E: oxygen-loaded SPIO@PFH-CHC nanodroplets and radiation therapy.

The 4T1 breast cancer cells were injected into mice at a concentration of 2 × 10^6^ cells/mice and waited 8 days. After 8 days, all mice were subjected to both oxygen-loaded SPIO@PFH-CHC nanodroplets and X-ray irradiation treatments on the 4th, 6th, 11th, 15th, and 18th days (sacrificed). Two parameters were monitored or measured for this in vivo animal test: the change in body weight and tumor volume.

The ratios of the mice’s body weights under treatments are shown in Figure 8B in which there were no significant differences between/among groups except on the last day, indicating that the combined treatment of oxygen-carrying nanodroplets and radiotherapy did not show substantial adverse effects on body weights.

Regarding the anti-tumor efficacy, the ratios of tumor volumes are shown in Figure 8C. Compared with the control group (ratio > 10 on the 18th day), the combined oxygen-loaded SPIO@PFH-CHC nanodroplets and radiotherapy reduced the tumor size considerably (ratio < 2.5 on the 18th day), though stand-alone treatment by either oxygen-loaded SPIO@PFH-CHC nanodroplets or radiotherapy is effective (both ratios ~5 on the 18th day).

The relatively high efficacy can be ascribed to DNA damages induced by radiotherapy and hydroxyl radicals produced by intracellular oxidative reactions from Fe^2+^ in SPIO@PFH-CHC nanodroplets. In addition, the similar tumor volumes from alone therapy imply an almost equal therapeutic effect of the X-ray irradiation and oxygen-loaded SPIO@PFH-CHC nanodroplets.

## 4. Some Quantitative Analyses

### 4.1. Interaction of Radiotherapy and SPIO@PFH-CHC Nanodroplets

Table 1 presents the data (survival percentage) from Figure 6B, the treatments of radiotherapy and nanodroplets against HeLa and 4T1 in the in vitro cytotoxicity test, and Figure 7B,C, the clonogenic assays. Based on these data, we can calculate the independence between the radiotherapy (RT) and nanodroplets (SPIO@PFH-CHC) with or without oxygen loading (denoted with or without @O_2_).

A popular approach to test the synergistic effect of a drug combination is to examine an average response of different combined doses to see whether the response value exceeds the Bliss prediction [27,32,33,34,35,36,37,38,39,40]. This prediction is centered around the *multiplicative* effect of two independent drugs.

Based on the probability theory, the combined effects can be interpreted as the statistical independence of two events of cell survival treated by drugs [27,34].

Consider that drugs *A* and *B* act independently. If the surviving fractions of cells or organisms under simultaneous and separate treatments are *S*(*A*∩*B*), *S*(*A*), and *S*(*B*), then the independence between *A* and *B* can be calculated as

(2) $$\frac{S\left(A\cap \;B\right)}{S\left(A\right)S\left(B\right)}$$

It is common to recognize that if this ratio is less than one, then there could be a synergy between *A* and *B*, whereas if it is larger than one, there can be antagonism between *A* and *B* [27,34]. It is worth noting that the synergy or antagonism is somewhat an indication of dependence (or interactions) between drugs *A* and *B*.

Table 2 shows the numerical values of the calculation. We notice that the combined effects of radiotherapy and nanodroplets for most cases are slightly larger than one. The clonogenic assays are more likely to be less than one except in cases under treatments of reoxygenation + RT and nanoparticles@O_2_ + RT.

As X-ray radiation can interact with nanodroplets, oxygen in culture media, and cells, the two treatments are not independent of each other. Equation (2) provides only a guiding answer to our cases. For the sake of brevity of this report, no further investigation will be pursued.

### 4.2. Copula Plot of Radiotherapy and SPIO@PFH-CHC Nanodroplets

**Figure 9:**
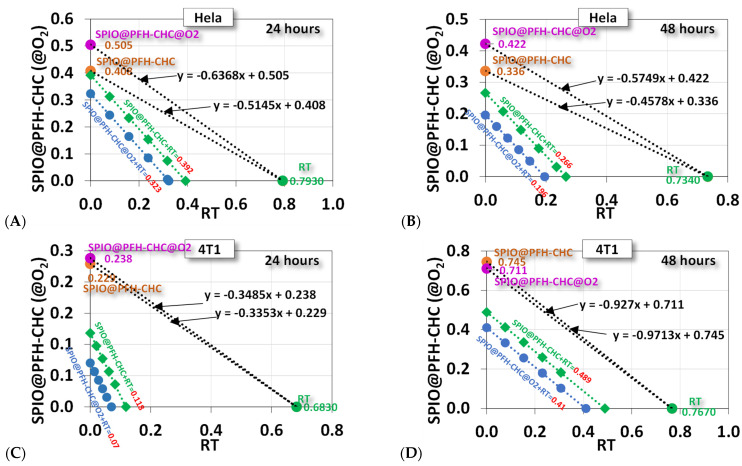
The copula of radiotherapy (RT) and SPIO@PFH-CHC nanodroplets (SPIO@PFH-CHC with or without oxygen loaded) from data of in vitro cytotoxicity assay shown in Figure 6B. The incubation is 24 and 48 h after X-ray irradiation (6 MV, 2 Gy). (**A**,**B**) are HeLa cervical cancer cells; (**C**,**D**) are 4T1 breast cancer cells. The concentrations of SPIO@PFH-CHC nanodroplets are 1600 μg/mL for HeLa cells and 800 μg/mL for 4T1 cells. For each figure, two isoboles were plotted for a linear combination of RT and SPIO@PFH-CHC or SPIO@PFH-CHC@O_2_.

Based on the data in Table 1, we can plot the copula of survival percentage in Figure 6B for the cytotoxicity tests of radiotherapy and nanodroplets against HeLa and 4T1 cells. Figure 9 shows the surviving percentages of HeLa cells and 4T1 cells at 24 and 48 h after X-ray irradiation. The horizontal axis is the survival of cells after radiotherapy alone, and the vertical axis is the treatment with nanodroplets of SPIO@PFH-CHC or SPIO@PFH-CHC@O_2_.

In each figure, we also include two isoboles for the combined treatment of SPIO@PFH-CHC + RT and SPIO@PFH-CHC@O_2_ + RT. The values of these two isoboles are respective experimental results drawn from Table 1. Readers can cross-examine these values in Table 1. We use a linear combination of the surviving percentages of single RT and single nanodroplets to calculate isoboles [39,40,41]. The linear combination is only a demonstration of possible outcomes, which nevertheless indicates that the combined treatment is quite effective because isoboles are fairly distant below the boundary of the RT-SPIO@PFH-CHC black dash line.

## 5. Conclusions

In this study, carboxymethyl hexanoyl chitosan (CHC) decorated with perfluorohexane (PFH) and superparamagnetic iron oxide (SPIO) nanoparticles (SPIO@PFH-CHC nanodroplets) was fabricated and investigated. The purpose of designing SPIO@PFH-CHC nanodroplets is to address the issue of hypoxia-induced radioresistance in cancer therapy. These SPIO@PFH-CHC nanodroplets have dual functionalities of oxygen carrying and radio enhancement effect via intracellular Fenton reactions with Fe^2+^ to produce hydroxyl radicals (HO^•^) and hydroxide ions (OH^−^), thus causing DNA damage.

The cell model is based on HeLa cervical cancer cells and 4T1 mouse breast cancer cells, and the animal model is 5-week-old nude female mice.

The experiments include serval steps to prove the functionalities of SPIO@PFH-CHC nanodroplets. (i) There is quick oxygen carrying by PFH in 5 min up to 30.13 ppm and slow oxygen release less than 25% of loaded oxygen over 8 h. (ii) Color changes in the tetramethylbenzidine assay (TMB + H_2_O_2_) reveal that the oxygen-loaded SPIO@PFH-CHC nanodroplets in PBS solutions favor an acidic environment (pH = 5.0) to oxidize TMB via the hydroxyl radicals HO^•^ produced by Fenton reactions. (iii) An immunostain on HeLa cells and 4T1 cells show that the oxygen-loaded SPIO@PFH-CHC nanodroplets can be uptaken by both cells in 6 h and help degrade the anti-hypoxia inducible factor 1 (HIF-1α) inside. (iv) The combined treatment on HeLa cells and 4T1 cells by oxygen-loaded SPIO@PFH-CHC nanodroplets and X-ray (6 MV, 2 Gy) shows higher DNA fragmentation in the sub-G1 phase and low turnout in the G2/M phase of cell cycles, indicating a synergism of SPIO@PFH-CHC nanodroplets and X-ray irradiation to damage DNA in both cancer cells. (v) An in vitro cytotoxicity assay shows that oxygen-loaded SPIO@PFH-CHC nanodroplets effectively suppress the cell viability of HeLa and 4T1 under the hypoxia condition. Even though 4T1 is more radioresistant than HeLa, oxygen-loaded SPIO@PFH-CHC nanodroplets still present decent potency. (vi) An in vivo mouse model indicates that the ratios of tumor-bearing mice’s body weights had no significant changes during 18 days of feeding under a serial treatment of oxygen-loaded SPIO@PFH-CHC nanodroplets and X-ray. However, the ratio of tumor volume on day 18 was substantially reduced to less than 2.5 times the volume on day 1. The animal model implies that treatment has minor side effects on mice.

Current experimental results exemplify that oxygen-loaded SPIO@PFH-CHC nanodroplets enable the reoxygenation of tumor tissues and can be an effective adjuvant for radiotherapy.

## Figures and Tables

**Table 1:** Survival percentage from the cytotoxicity test against HeLa cells and 4T1 cells in Figure 6B and clonogenic assays in Figure 7B,C.

Hela Cells	Treatment						
	RT	SPIO@PFH-CHC	Reoxygenation	Reoxygenation + RT	SPIO@PFH-CHC@O_2_	SPIO@PFH-CHC + RT	SPIO@PFH-CHC@O_2_ + RT
**Cytotoxicity (24 h)**	0.793	0.408			0.505	0.392	0.323
**Cytotoxicity (48 h)**	0.734	0.336			0.422	0.266	0.196
**Clonogenicity**	0.484	0.701	1.072	0.424	0.704	0.339	0.232
**4T1 Cells**	**Treatment**						
	**RT**	**SPIO@PFH-CHC**	**Reoxygenation**	**Reoxygenation + RT**	**SPIO@PFH-CHC@O_2_**	**SPIO@PFH-CHC + RT**	**SPIO@PFH-CHC@O_2_ + RT**
**Cytotoxicity (24 h)**	0.68	0.229			0.238	0.118	0.07
**Cytotoxicity (48 h)**	0.77	0.745			0.711	0.489	0.41
**Clonogenicity**	0.84	0.79	0.913	0.821	0.751	0.34	0.256
**Remark**	RT: Radiotherapy						
	@O_2_: Under reoxygenation effect by degrading oxygen-loaded SPIO@PFH-CHC						

**Table 2:** The ratio of *S*(*A*∩*B*)/*S*(*A*)*S*(*B*) from the cytotoxicity tests (24 and 48 h) against HeLa cells and 4T1 cells in Figure 6B and clonogenic assays in Figure 7B,C.

Hela Cells	S(RT∩SPIO@PFH-CHC)/(S(RT) × S(SPIO@PFH-CHC))	S(Reoxygenation∩RT)/(S(RT) × S(Reoxygenation))	S(RT∩SPIO@PFH-CHC@O_2_)/(S(RT) × S(SPIO@PFH-CHC@O_2_))
**24 h**	1.2116		1.5608
**48 h**	1.0786		1.7111
**Clonogenic**	0.9992	0.817195017	2.0750
**4T1 Cells**	**S(RT∩SPIO@PFH-CHC)/(S(RT)** **× S(SPIO@PFH-CHC))**	**S(Reoxygenation∩RT)/(S(RT)** **× S(Reoxygenation))**	**S(RT∩SPIO@PFH-CHC@O_2_)/(S(RT)** **× S(SPIO@PFH-CHC@O_2_))**
**24 h**	0.7544		1.5217
**48 h**	0.8558		1.2443
**Clonogenic**	0.5148	1.075637915	1.1371

## Data Availability

The original contributions presented in this study are included in the article. Further inquiries can be directed to the corresponding authors.
